# A Review of the Preparation of Porous Fibers and Porous Parts by a Novel Micro-Extrusion Foaming Technique

**DOI:** 10.3390/ma17010172

**Published:** 2023-12-28

**Authors:** Zelin Wang, Hanyi Huang, Yushu Wang, Mengnan Zhou, Wentao Zhai

**Affiliations:** School of Materials Science and Engineering, Sun Yat-sen University, Guangzhou 510275, China; wangzlin7@mail2.sysu.edu.cn (Z.W.); huanghanyi@sysunc.com (H.H.); wangysh56@mail2.sysu.edu.cn (Y.W.); zhoumn@mail2.sysu.edu.cn (M.Z.)

**Keywords:** porous fibers, porous parts, physical foaming, MEF

## Abstract

This review introduces an innovative technology termed “Micro-Extrusion Foaming (MEF)”, which amalgamates the merits of physical foaming and 3D printing. It presents a groundbreaking approach to producing porous polymer fibers and parts. Conventional methods for creating porous materials often encounter obstacles such as the extensive use of organic solvents, intricate processing, and suboptimal production efficiency. The MEF technique surmounts these challenges by initially saturating a polymer filament with compressed CO_2_ or N_2_, followed by cell nucleation and growth during the molten extrusion process. This technology offers manifold advantages, encompassing an adjustable pore size and porosity, environmental friendliness, high processing efficiency, and compatibility with diverse polymer materials. The review meticulously elucidates the principles and fabrication process integral to MEF, encompassing the creation of porous fibers through the elongational behavior of foamed melts and the generation of porous parts through the stacking of foamed melts. Furthermore, the review explores the varied applications of this technology across diverse fields and imparts insights for future directions and challenges. These include augmenting material performance, refining fabrication processes, and broadening the scope of applications. MEF technology holds immense potential in the realm of porous material preparation, heralding noteworthy advancements and innovations in manufacturing and materials science.

## 1. Introduction

**Figure 1:**
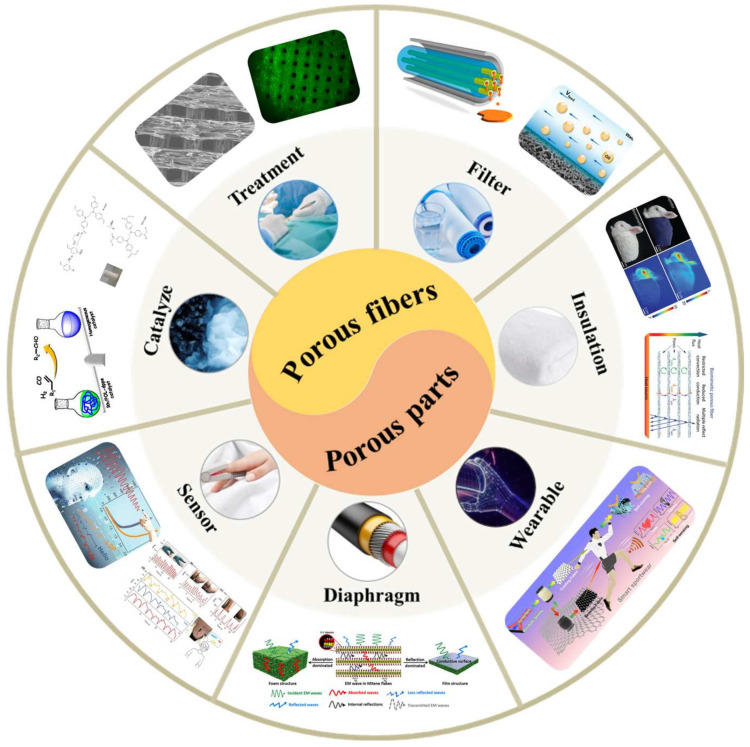
The application of porous fibers and porous parts in fields such as filtration [36], insulation [13], wearability [37], diaphragms [38], sensors [39], catalysis [40], and medical treatment [41].

Porous materials featuring precisely defined and interconnected porous structures have attracted considerable attention across diverse fields owing to their distinctive properties and versatile applications [1,2,3]. The distinctive nature of porous structures arises from their fundamental characteristics, including a high surface area-to-volume ratio, interconnected pore networks, and a controllable pore size distribution [4,5]. Within the realm of porous polymer materials, porous fibers and porous parts emerge as two distinct morphological entities characterized by different dimensional attributes, yet they are inherently interconnected. Porous fibers denote materials composed of fibers exhibiting a porous structure, whereas porous parts constitute integral components or materials with pores or porous structures throughout their overall composition [6]. Porous fibers highlight the pore structure at the scale of individual fibers, whereas porous parts exhibit greater versatility, serving as integral components of diverse shapes, and find application across a broader spectrum of uses. This association stems from the broader understanding that porous fibers can be considered a subset of porous parts. This categorization is justified by the transformation of porous fibers into the latter category through processes such as stacking, weaving, and combining. Serving as significant subcategories, both porous fibers and porous parts exhibit extensive potential for diverse applications [7]. They exhibit exceptional functional properties, including superior adsorption [8,9], permeation separation [10,11,12], thermal insulation [13,14], hydrophobic properties [15,16,17], and catalytic properties [18,19,20,21]. Consequently, they find widespread utilization in various domains such as supercapacitors [22], drug delivery systems [15,23], wastewater purification [5,24], thermal insulation materials [25], sound absorption materials [26,27], sensors [28,29], and tissue engineering [30,31], as shown in Figure 1. Significantly, these subclasses face shared challenges, including the regulation of pore structure, material selection, and fabrication methods. These challenges play pivotal roles in the entire preparation process of porous materials. Through a comprehensive grasp and application of the principles governing the functional applications of porous structures, researchers can adeptly customize and regulate characteristics such as porosity, pore size, and pore distribution to meet specific requirements [32,33,34,35]. This enables a deeper exploration and refinement of the design, fabrication, and utilization of porous materials.

Various fabrication methods have been developed and applied in the preparation of porous structures to cater to different application requirements. A fiber is a slender material with a high aspect ratio, which can be derived from natural sources, including plant fibers (such as cotton and linen) or animal fibers (like wool and silk) or artificially synthesized fibers, as in the case of synthetic fibers (such as nylon and polyester) [42]. Porous fibers are characterized by the presence of numerous small pores or voids within the fiber material. These pores can exhibit various scales, ranging from microscopic and nanoscale to macroscopic at the millimeter level [43]. The manufacturing methods of porous fibers have been closely associated with spinning techniques [44,45,46,47,48]. In 2010, Yang et al. [49] prepared porous polyamide (PA) fibers by dry spinning using a solution of PA dissolved in a formic acid/chloroform co-solvent. The mechanical properties of the resultant fibers were investigated, revealing that the development of the porous structure could be attributed to the evaporation of the low-boiling-point solvent during the spinning process. In recent years, advancements in equipment and deepening research have given rise to new methods for fabricating porous fibers, garnering significant attention. Notably, high internal phase emulsion template methods [50,51,52], coaxial wet spinning [25,53], freeze spinning [6,13,54], and microfluidic spinning [37,55] have gained prominence. Among them, wet spinning refers to a molding method where the spinning solution is extruded into a coagulation bath through a syringe, resulting in the solidification of the polymer into porous fibers through a double-diffusion process. These methods share a common strategy for fabricating porous fibers, involving the initial formation of fibers in a solvent, followed by post-processing to remove the second phase and obtain the desired pore structure. This implies that their fabrication processes involve the use of significant amounts of organic solvents, challenging conditions for controlling the reaction, stringent environmental requirements, and complex post-processing procedures. Recent studies propose the integration of electrospinning with gas dissolution foaming to achieve porous fibers at the micrometer or even nanometer scale [56,57]. This approach harnesses the benefits of spinning technology while minimizing solvent usage. Porous parts share similar concepts of pore formation with porous fibers. The frequently used preparation methods include the high internal phase emulsion template method [58,59], solvent etching [60], freeze-drying [61,62], and thermally induced phase separation [63]. Foaming technology stands out as a commonly employed method for the preparation of porous materials, accomplishing lightweighting and the formation of closed-cell structures by introducing gas bubbles into the material [64,65]. Nevertheless, these methods are limited to generating sheet- or block-shaped porous materials and are accompanied by environmental concerns and complexity. In general, the preparation and production of porous fibers and parts encounter substantial challenges concerning environmental sustainability and economic viability. The efficient and continuous execution of production operations, as well as the creation of complex three-dimensional porous parts, have prompted researchers to explore novel methods that are both simple and environmentally friendly while maintaining high operability.

**Figure 2:**
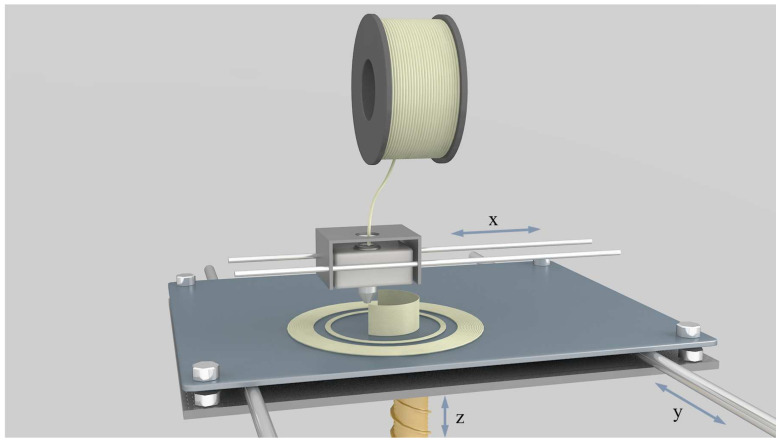
Schematic representation of the FDM process.

Over the past three decades, 3D printing, also recognized as Additive Manufacturing (AM), has made substantial advancements and is currently deployed across diverse fields [66,67]. For the manufacturing of structurally complex components, AM provides an economical production solution instead of traditional processes [68,69]. Fused Deposition Modeling (FDM) is one of the most widely used 3D printing technologies, employing a layer-by-layer deposition approach [70]. The most commonly used implementation process in this technology is the filament extruder [71]. When printing and forming, thermoplastic material is extruded in a filament from a nozzle and is deposited layer by layer on the build platform. Each layer is rapidly cooled and solidified to achieve precise printing, as illustrated in Figure 2. Once a layer is printed, the construction platform will descend, and subsequent layers will be deposited and adhered to the top of the previous layer [71]. Researchers have previously integrated 3D printing with fabrication methods for porous parts, leading to the emergence of innovative manufacturing processes [72,73,74]. For example, Choi et al. [41] combined the chemical foaming method with FDM technology to achieve in situ foaming and the one-step formation of lightweight (polylactic acid) (PLA) parts. This approach simplifies the fabrication of graded porous parts and provides high shape freedom for porous structures. Furthermore, Li et al. [75] have recently introduced a novel method for preparing porous fibers by combining foaming technology with the FDM process. This approach has demonstrated success while concurrently addressing environmental protection considerations and enhancing production efficiency. In the last decade, driven by increasing environmental awareness, foaming technology has undergone a shift toward adopting green and sustainable practices. Technologies such as water-based foaming and supercritical fluid foaming have gained prominence, reducing reliance on organic solvents and minimizing environmental pollution. These advancements present new avenues for the manufacturing of porous fibers and porous parts. MEF belongs to environmentally benign physical foaming techniques. It has emerged as a new method in recent years to expand the manufacturing capabilities of porous fibers and porous parts, becoming a hot research trend in the foaming field.

**Figure 3:**
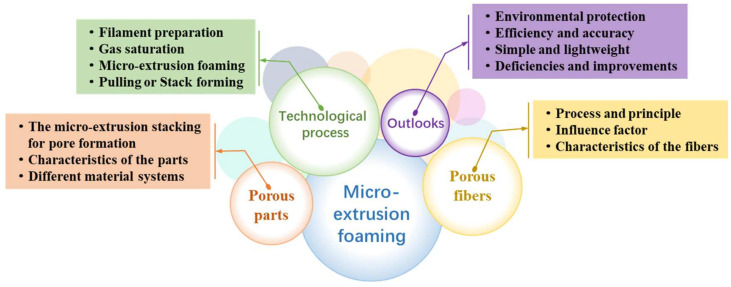
MEF frame diagram.

In response to environmental concerns, challenges related to complex processing, and low fabrication efficiency in the preparation of porous polymer fibers and parts, a novel solution has been proposed and developed: Polymer High-Pressure Fluid MEF technology. In contrast to alternative processes and technologies, this method achieves a green, environmentally friendly, highly efficient, and scalable preparation process. MEF stands out as a novel method for the preparation of porous fibers and parts, amalgamating the benefits of physical foaming while drawing inspiration from FDM technology. This technique involves impregnating a compressed gas into the polymer melt and inducing cell nucleation and growth during the melt extrusion process, resulting in porous structures. The aim of this review is to furnish a comprehensive overview of the process principles underlying MEF technology, coupled with a synthesis and outlook on recent research developments. The content is structured into four main segments. Firstly, the review introduces the overall process of MEF technology, covering its classification, front-end preparation, and fundamental principles. Secondly, two distinct sections are dedicated to the discussion of options for fabricating end products, specifically porous fibers and porous parts. Lastly, the review outlines the prospects for future research concerning the preparation of porous fibers and parts via MEF, as depicted in Figure 3.

## 2. MEF

### 2.1. Polymer Physical Foaming Technology

**Figure 4:**
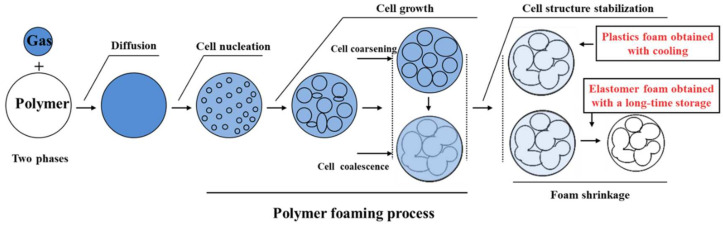
Evolution law of cells during the process of polymer foaming [64].

MEF is a type of melt foaming technique in which the foaming melt undergoes controlled heating to achieve complex structures by regulating its residence time in the micro-extrusion die. Categorized as a physical foaming technique for polymers, the resulting porous polymer fibers and parts fall within the domain of microcellular polymers. The notion of microcellular polymers or polymer microcellular foamed materials was initially introduced by Professor Suh and colleagues at the Massachusetts Institute of Technology (MIT) in the 1980s [76]. Subsequently, Suh et al. proposed the foundational approach of employing physical foaming techniques for the preparation of microcellular polymers. Polymer physical foaming techniques entail the utilization of high-pressure fluids or supercritical fluids, such as CO_2_ and N_2_, as foaming agents to generate foamed polymer materials. These high-pressure fluids and supercritical fluids, serving as foaming agents, are characterized by their non-toxic and non-polluting nature, lack of residue, and diverse sources. This affords them the advantage of being environmentally friendly throughout the processing and utilization phases. The evolution of cell morphology during the polymer physical foaming process is depicted in Figure 4 and can be segmented into four stages [64]: (1) formation of homogeneous polymer/fluid system under certain saturation conditions via a gas diffusion process, (2) inducing cell nucleation by either raising the temperature or rapidly depressurizing the system to achieve supersaturation, (3) growth and coalescence of cells, and (4) rapid cooling of the foamed sample to stabilize the cellular structure.

Owing to heightened environmental concerns and increased awareness, the physical foaming technique for polymers has garnered significant attention and witnessed substantial development since its inception. Various foaming techniques have been developed for manufacturing plastic and elastomeric foams, with the main ones being batch foaming [65,77,78], injection molding foaming [65,79,80], and continuous extrusion foaming [65,81,82]. In recent years, in pursuit of shape-unrestricted polymer foams, polymer physical foaming technology has been employed in 3D printing, giving rise to the emergence and advancement of MEF. The foaming processes of the four techniques share similarities, albeit with some distinct differences in their characteristics. Table 1 provides a comparison and summary of the general characteristics of these four foaming techniques [64]. MEF and continuous extrusion foaming share similar extrusion foaming processes, but their primary distinction lies in both the extrusion foaming process and the back-end preparation methods.

### 2.2. Concise Overview of MEF Process and Principles

While continuous extrusion foaming technology has been extensively documented in both academic and industrial settings, it grapples with challenges related to fabricating minute cell structures and the intricacies of controlling three-dimensional structures. These challenges limit its efficacy in producing porous fibers and diverse-shaped porous parts. MEF technology effectively addresses these issues. On one hand, the MEF process avoids screw shearing or high-pressure extrusion, enabling it to yield superior porous fibers in terms of both structure and performance compared to continuous extrusion foaming. On the other hand, MEF integrates the 3D printing technology of FDM, imparting high design flexibility for 3D structures during the molding process.

**Figure 5:**
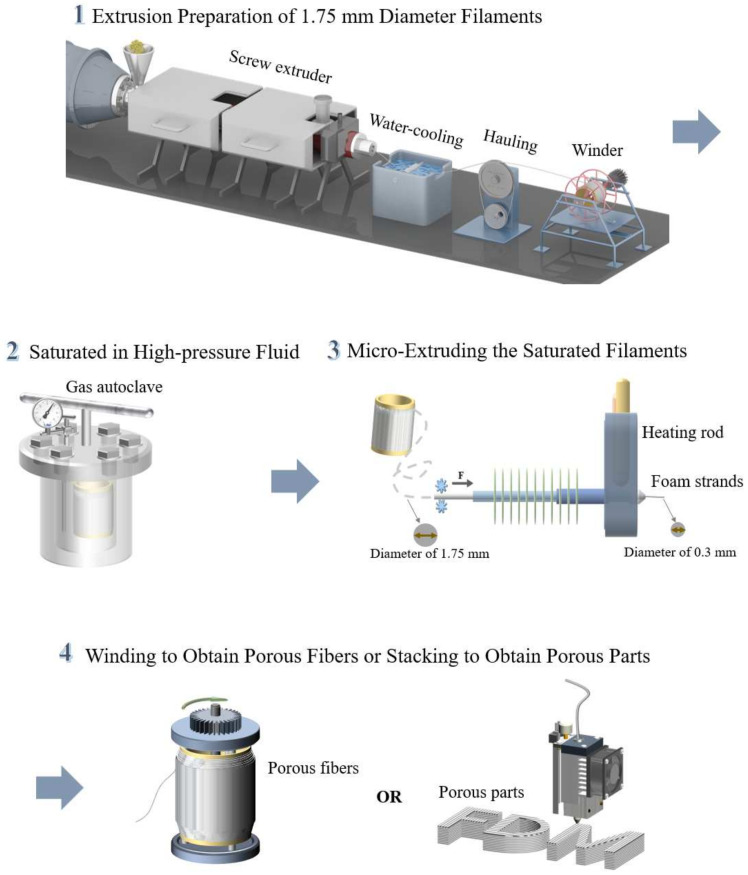
Implementation process of MEF technology.

Figure 5 offers a comprehensive illustration of the MEF process, with specific emphasis on the extrusion foaming of polymer filaments and the generation of porous fibers and parts. The MEF technology process comprises four key steps: Extrusion Preparation of 1.75 mm Diameter Filaments, Saturation in High-pressure Fluid, Micro-Extruding of the Saturated Filaments, and Winding to Obtain Porous Fibers or Stacking to Obtain Porous Parts. These stages are detailed as follows.

#### 2.2.1. Extrusion Preparation of 1.75 mm Diameter Filaments

Polymer extrusion processes, as a thoroughly established and mature traditional manufacturing method, have attained a high level of proficiency. Employing a front-end screw extruder coupled with a rear winding machine and air/water cooling devices, the process consistently produces stable filaments with a diameter of 1.75 mm. Polymer filament diameters within allowable tolerances, with an error not surpassing 0.05 mm, are adequate to meet the requirements of the majority of FDM 3D printers available in the market [83].

#### 2.2.2. Saturated in High-Pressure Fluid

Similar to batch foaming techniques, prior to the MEF of the extruded polymer filaments, the samples need to be saturated with high-pressure fluid foaming agents such as CO_2_ or N_2_ [84,85]. High-pressure fluids demonstrate outstanding penetration capabilities, particularly in a supercritical state. These fluids exhibit dissolving properties akin to liquids, coupled with a gas-like viscosity and diffusion coefficients, rendering them soluble in the majority of polymers [86]. To prevent induced foaming inside the high-pressure vessel during the pressure relief process, the saturation temperature is usually set much lower than the softening temperature of the polymer (typically between 25 °C and 60 °C), and the saturation pressure should not be too high (typically between 3 MPa and 10 MPa). The direct mixing of non-gaseous foaming agents (such as chemical foaming agents, expandable microspheres, glass microspheres, etc.) in the screw extruder can also achieve foaming effects, which imposes more specific requirements on the extrusion process. Furthermore, utilizing a high-pressure fluid as the foaming agent offers significant advantages in terms of environmental sustainability. This choice aligns with the mainstream trend in academic research and industrial production, making it a more promising and environmentally friendly option [87].

#### 2.2.3. Micro-Extruding of the Saturated Filaments

After a sufficient saturation time, the polymer filaments are taken out from the high-pressure vessel and subjected to micro-extrusion equipment, resulting in foaming and forming porous stranded structures through temperature-induced melt foaming. Various parameters, including high-pressure fluid solubility, filament feed rate, nozzle temperature, nozzle diameter, and cooling fan speed, among others, must be meticulously considered and harmonized during this process. Optimal parameter settings contribute to achieving a uniform cell structure and size. In contrast, lower high-pressure fluid solubility, slower filament feed rates, and higher nozzle temperatures may result in larger cells and uneven structures. Careful control and coordination of these parameters are crucial for the successful implementation of the MEF process [85,88,89]. Typically, a nozzle with a diameter of 0.3 mm is employed to extrude the molten strand. This choice is informed by the fact that, as the nozzle diameter decreases, the extrusion speed of the foam strand increases, and the extrusion port encounters higher pressure from the material viscosity. This phenomenon is akin to a small nozzle on a water gun. Consequently, the nozzle diameter cannot be smaller than a certain value, usually 0.3 mm, to avoid blockage at the extrusion outlet. On the contrary, a larger nozzle diameter demands more heat exchange, necessitating higher temperatures. This increased temperature could potentially compromise the porous structure of the strand, proving detrimental to subsequent winding or stacking forming processes.

#### 2.2.4. Winding to Obtain Porous Fibers or Stacking to Obtain Porous Parts

Following the Micro-Extruding of the Saturated Filament process, researchers have two options for processing the foamed strands: winding or stacking, with the aim of producing porous fibers or porous parts. While both winding and stacking introduce additional complexities in the subsequent processes, they can be comprehensively understood and controlled to achieve desirable end products. Undoubtedly, the back-end processing no longer functions as a singular directive but presents more opportunities and avenues for the development of MEF.

**Figure 6:**
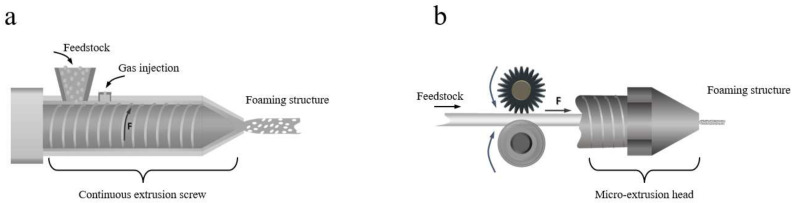
The extrusion foaming process of continuous extrusion foaming (**a**) and MEF (**b**).

Now, a further comparison between continuous extrusion foaming and MEF can be made. Figure 6 illustrates the extrusion foaming processes for both methods. In the continuous extrusion foaming process, high-pressure fluid, acting as the physical foaming agent, is injected into the screw cavity of the extruder through the injection system and forms a homogeneous polymer/fluid system. The homogeneous melt is extruded out of the die of the extruder, causing a dramatic pressure dropping, which induces nucleation and then the growth of bubbles. The combination of high-pressure mixing and a sudden pressure drop can readily lead to the coalescence, rupture, and annihilation of internal cells within the foam melt. This phenomenon results in an uneven cell size and distribution, a sparse cell structure, and other varied structural morphologies in the final product [75,90,91,92,93,94,95]. To improve the cell morphology in continuous extrusion foaming, it is necessary to control complex parameters to enhance cell nucleation with nanoparticles and to restrict cell coalescence by increasing the melt strength of the polymer [93,96,97]. However, this process typically faces limitations in the preparation of foamed fiber because of the presence of a high melt strain and a large bubble size within the polymer melt [98,99]. In contrast, MEF relies on the propulsion of the filament within the channel by gears rather than the shear forces of the screw [100]. Additionally, the saturation of the foaming agent in the polymer occurs before the extrusion foaming process, eliminating the need for shear mixing of the polymer and foaming agent by the screw. This enables MEF to achieve a more dense and uniform cellular structure [101,102,103].

## 3. MEF for Porous Fibers

### 3.1. Process and Principle

**Figure 7:**
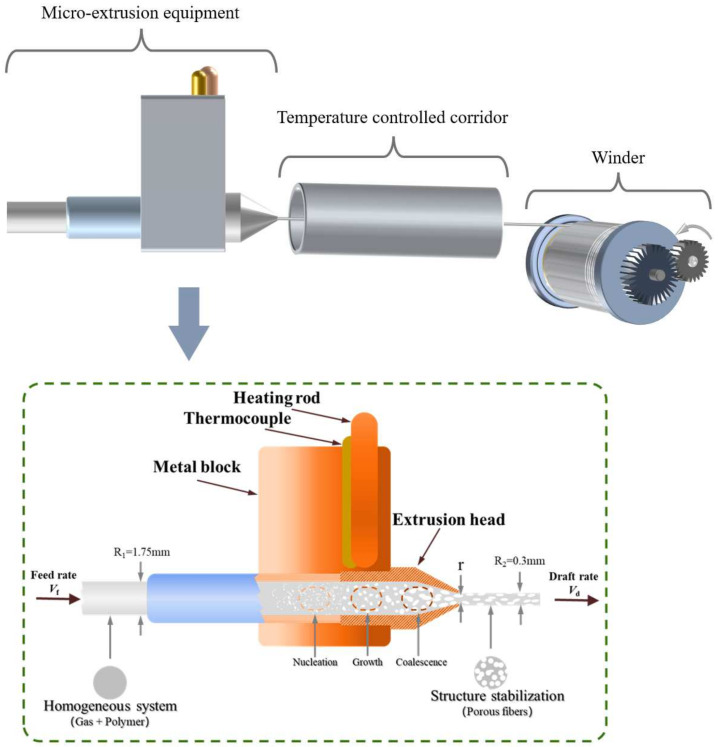
Schematic diagram of the mechanism of MEF for fiber preparation.

Figure 7 illustrates a schematic diagram of the preparation of porous fiber and its mechanism using MEF. The essential components for the preparation of porous fibers using MEF encompass the micro-extrusion equipment, the temperature-controlled corridor, and the winder. The micro-extrusion equipment provides the foaming conditions, where the fibers develop their porous structure. The temperature-controlled corridor ensures the rapid solidification or continuous growth of the cells, and the winder stretches the foam strand, collecting them to form the final product (porous fibers). In the lower section of Figure 7, a detailed depiction is provided of the internal structure of the micro-extrusion equipment and the foaming process of the polymer/gas filaments. The polymer/gas mixture filament with a diameter of 1.75 mm is fed through the gear set to the micro-extrusion nozzle for foaming. The temperature at the micro-extrusion nozzle is intentionally maintained higher than the melting temperature of the polymer. This ensures that the polymer/gas filament can rapidly melt and be extruded to form the foam strand within the heating unit.

When passing through the interior of a metal block at a specific temperature, the polymer exhibits low thermal conductivity, and the heat transfer from the outside to the inside takes some time. Therefore, within the micro-extrusion head, the polymer/gas filament undergoes three distinct states: the glassy state, the high-elastic state, and the viscous flow state [75,104,105]. The glass transition of the polymer/gas filament occurs at a distance from the extrusion head. When the polymer/gas mixture is in a glassy state, the formation of cell nuclei is limited. In the high-elastic state, nucleation and the growth of cells within the matrix occur. As the temperature continues to rise, and the polymer/gas mixture enters the viscous flow state, the cell growth and coalescence become more pronounced. In summary, the polymer/gas mixture undergoes three state changes accompanied by the nucleation, growth, and coalescence processes of the cells in Figure 7, ultimately evolving into porous fibers after extrusion and drafting.

While passing through the extrusion head, the extruded foaming melt undergoes a certain pressure due to the foaming process. However, owing to the uniaxial tensile force, the extrusion swelling of the foaming melt at the nozzle exit is nearly negligible. Consequently, the primary factor influencing the change in fiber diameter is the volume expansion resulting from foaming. It is worth mentioning that fiber diameter is also one of its important characteristics. In the process of preparing porous fibers through MEF, the nozzle diameter determines the final diameter of the fibers. The smaller the nozzle diameter, the finer the fibers will be. However, due to the limitations of foaming and the requirement of stretching for the strength of polymer melt, the current technology limits the diameter of MEF fibers to between 1.0 and 0.1 μm [75,106]. MEF is a rapid heating-induced foaming technique for cell nucleation, and it is evident that the main difference compared to heated foaming is that the polymer here undergoes a melting process. MEF involves a complex process of polymer melting, and the cells undergo nucleation and secondary derivations within the melt. Therefore, controlling the time the melt spends in the micro-extrusion nozzle is crucial. In other words, the limited time for cell growth and coalescence is the fundamental reason for the successful preparation of porous fibers.

### 3.2. Influence Factors of Porous Fiber Fabrication

**Figure 8:**
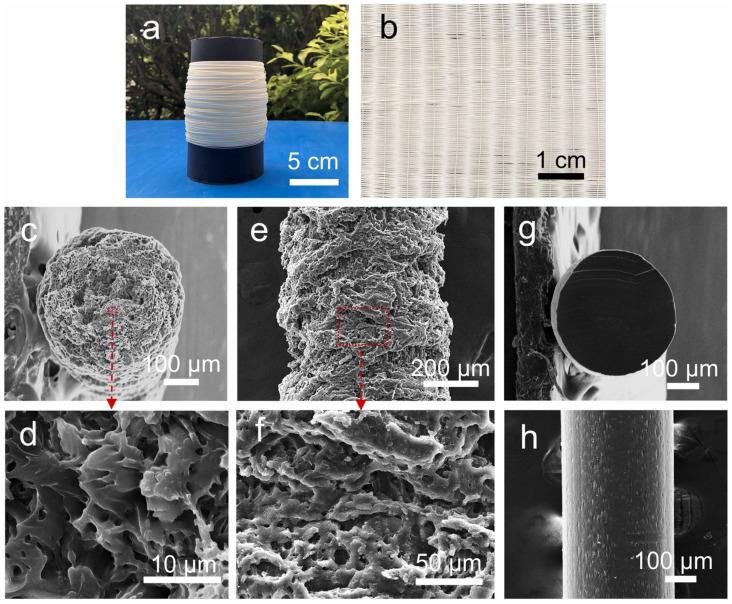
Porous PEI fibers obtained from MEF. (**a**) Optical image of a roll of the porous PEI fibers. (**b**) Optical image of the porous PEI fiber textile. SEM images of the cross-sectional (**c**,**d**) and surface (**e**,**f**) morphology of the porous PEI fibers, as well as SEM images of the cross-sectional (**g**) and surface (**h**) morphology of PEI fibers extruded from solids [91].

Based on our preceding discussion, the fabrication of porous fibers using the MEF method is not only theoretically feasible but also holds great promise for practical applications. Li et al. [90] first used the MEF method to prepare porous polyetheretherketone (PEEK) fibers and achieved success. They utilized PEEK’s low gas diffusion rate and high T_g_ to manufacture porous PEEK fibers with internal micropores and rough surface structures, and they studied the effects of different process parameters on the pore morphology of porous PEEK fibers. MEF relies on the supercritical fluid dissolved in the polymer as the foaming factor, temperature triggering induces foaming nucleation, and time limiting controls the morphology of the pores, so the main influencing factors are the following five points: (1) saturation pressure, (2) saturation temperature, (3) desorption time, (4) foaming temperature, and (5) drawing rate. But in reality, the first three factors can in turn be combined into one point: the gas content dissolved in the polymer because the ultimate orientation of their changes is the same. With CO_2_ gas, a higher saturation pressure, either a lower saturation temperature or a shorter desorption time, leads to an increase in its solubility in the polymer. According to classical nucleation theory, a high gas content in the polymer creates a large pressure differential, which creates a greater driving force for cell nucleation [107,108,109]. This will result in an increase in nucleation sites and a decrease in the critical size of nucleation, obtaining a denser and more uniform porous structure. At the same time, the increase in nucleation rate leads to the rapid completion of the foaming process, and the size of the foam cells correspondingly decreases. On the contrary, low gas content will have the opposite effect. The above rules have been clearly confirmed for microscale cells, while the sensitivity of nanoscale cells to gas solubility is much smaller [110,111,112]. Zhou et al. [91] also prepared porous polyetherimide (PEI) fibers (Figure 8) using the MEF method, and due to foaming expansion, the fiber diameter was slightly larger than the nozzle diameter (0.4 μm). They studied the cell density and size of porous fibers under different saturation pressures and observed similar phenomena as mentioned above. The foaming temperature refers to the real-time temperature of the micro-extrusion nozzle, which is measured by a thermocouple placed at the heating metal block center. Increasing the foaming temperature increases the thermodynamic instability of the system and reduces the viscosity of the polymer melt, thereby promoting cell growth and coalescence. Therefore, the cell size of porous fibers increases with the increase in the foaming temperature, while the cell density decreases. The drawing rate is a key process parameter for collecting porous fibers into rolls, which affects the growth and final shaping of cells within the fibers. The uniaxial stretching force applied to the extruded fiber by drawing increases its elastic strain energy during foaming, which not only reduces the energy barrier of cell nucleation but also provides a driving force for the formation of the cell nucleus. In addition, the magnitude of uniaxial tensile force is generally positively correlated with the nucleation rate of cells.

In summary, under the prerequisite of a certain high-pressure fluid content in the material, the temperature and time constraints (drawing rate) are the primary factors influencing the size and distribution of cells. Undoubtedly, the temperature must be sufficiently high to ensure that the material forms a foaming melt. At this point, there is a temperature foaming window, where the higher the temperature, the faster the cell growth and coalescence, and the larger the cell size, the lower the density. When the temperature exceeds this window, an inadequate melt viscosity prevents extrusion and drawing, potentially leading to degradation. Furthermore, time constraints are a critical factor in cell growth. In the micro-extrusion head, growth, coalescence, and uniform dense distribution of cells require a certain amount of time, albeit brief (around 1 s, with minor variations among different materials) [75]. If the residence time of the foaming melt in the micro-extrusion head exceeds this duration, pores will coalesce dramatically, resulting in uneven sizes and distributions.

### 3.3. Characteristics of the Fibers

**Figure 9:**
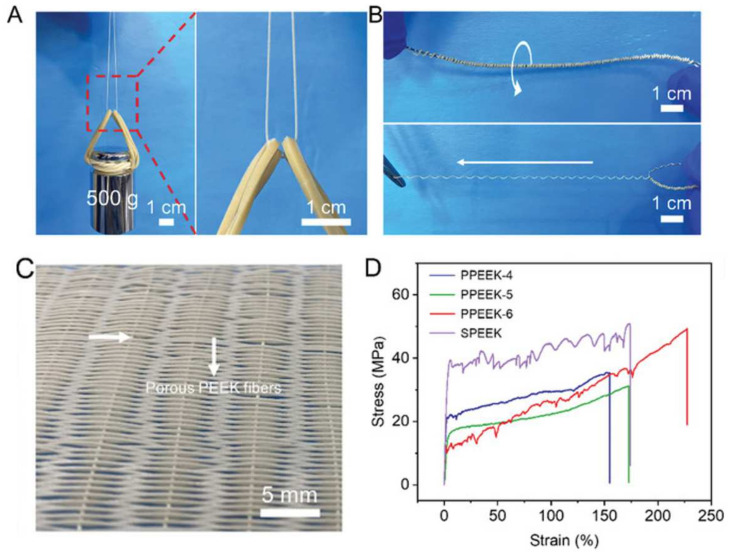
Weaving and mechanical tensile properties of PEEK porous fibers [75]. (**A**) Optical image of porous PEEK fibers with the diameter of 0.39 ± 0.022 mm hanging with 500 g weights. (**B**) Optical image of porous PEEK fibers wound around a 13 cm-long wire. (**C**) Optical image of textile woven with porous PEEK fibers. (**D**) Stress-strain curve.

**Figure 10:**
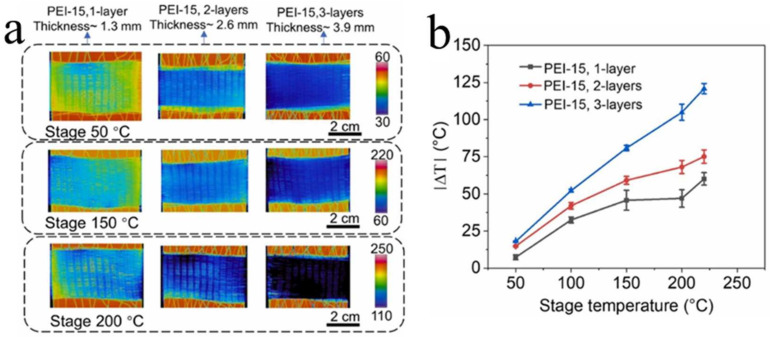
Thermal insulation of PEI porous fiber woven fabric [91]. (**a**) Infrared images of PEI textiles with different layers placed on a hot plate. The temperature of the fabric surface was read from the corresponding infrared images as the stage temperature varied between 50 °C and 220 °C. (**b**) Plot of the ǀΔ*T*ǀ versus stage temperature for different layers of the PEI textile.

Multifunctional porous fibers with excellent mechanical properties have received widespread attention in the fields of personal thermal management textiles and intelligent wearable devices [25]. The porous fibers prepared by MEF not only have high production efficiency (up to 10.5 cm/s) but also have diverse cell structures and excellent weaving properties [90]. The group of Zhai [75,91] explored a series of properties of physically foamed PEEK and PEI porous fibers, demonstrating their potential for application in the textile field. In Figure 9, they show that a single PEEK fiber with a diameter of only 0.39 (±0.022) mm can stably withstand a weight of 0.5 kg and a tensile strain of up to 234.8%. PEI fibers also have excellent weaving performance, with a maximum temperature difference of over 50 °C for their single-layer textiles in terms of their thermal insulation, as shown in Figure 10. This is due to the sub-microporous structure on the surface and the inside of the fiber, which changes the heat transfer mechanism of the textile. The preparation of porous fibers through MEF has not yet been applied in material systems such as elastic polyurethane, degradable polylactic acid, and polyethylene, so its subsequent development has attractive prospects. In summary, the emergence of the first porous PEEK and PEI physically foamed fibers has opened up new avenues for expanding applications and improving production efficiency.

## 4. MEF for Porous Parts

### 4.1. The Micro-Extrusion Stacking for Pore Formation

In the additive manufacturing process of FDM, micro-extrusion stacking provides the required extrudates for constructing three-dimensional plastic parts and facilitates the final material forming [113]. Applying purposeful control to this process to create pores and obtain porous plastic parts to meet human needs in various fields such as lightweight manufacturing [114,115,116], energy absorption [117,118,119], insulation and flame retardancy [120,121], and medical scaffolds [122,123,124,125] is becoming an emerging strategy. At present, the methods of micro-extrusion melt stacking for manufacturing porous parts reported in the literature can be divided into four categories, namely micro-extrusion foam stacking (in situ foaming), microsphere doping, post-batch foaming, and ordered cell unit printing [31,121].

**Figure 11:**
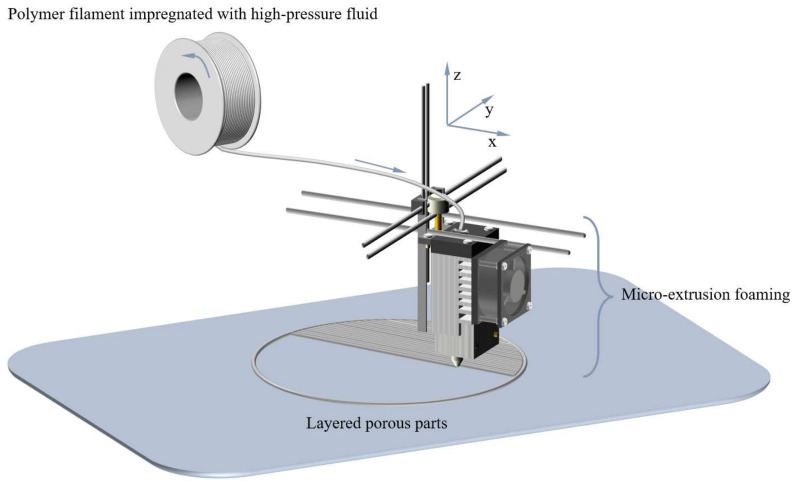
Micro-extrusion foam stacking process for preparing porous parts.

Micro-extrusion foam stacking integrates FDM technology and physical foaming technology, achieving continuous and efficient manufacturing of porous parts. This process is shown briefly in Figure 11, which likewise contains the indispensable micro-extrusion head. The foaming strands extruded from the nozzle of the micro-extrusion head remain in a viscous state for a short time and are stacked layer by layer along the *x*, *y*, and *z* axes and bond with high accuracy under program control. Since polymer filament is already saturated with high-pressure fluids, the foaming process of cell nucleation, growth, and coalescence also occurs when it passes through the heated micro-extrusion head. Obviously, the foam strand used for stacking is actually the precursor of porous fibers (i.e., non-stretched porous fibers), so parameters such as extrusion rate, temperature, and desorption time have similar effects on the cell size and density of porous parts. Previously, PEI was used for MEF to prepare porous fibers, demonstrating its excellent foaming behavior. Afterward, Zhou et al. [126] used PEI for micro-extrusion foam stacking to prepare porous parts. Research showed that as the saturation pressure decreased, desorption time was prolonged, nozzle temperature increased, cell size gradually increased, and cell density gradually decreased. This fully demonstrates the bidirectional application potential of MEF for the same material in the preparation of porous fibers or porous parts. It is worth mentioning that if supercritical fluid saturation is replaced with chemical foaming agent doping, porous parts can also be obtained through micro-extrusion foam stacking [41,116]. However, this often introduces issues such as toxic foaming agents, a mismatch between foaming agent decomposition temperature and polymer processing temperature, residual foaming agent decomposition products, and increased processing costs.

In addition, there are three other methods for manufacturing porous parts through micro-extrusion stacking, namely microsphere doping, post-batch foaming, and ordered cell unit printing [101,127,128]. The porous parts formed by micro-extrusion and stacking after the microspheres are doped into the polymer filament are also called syntactic foams [129,130,131]. The formation of its porous structure depends on the hollow pores of the glass microspheres or the thermal expansion effect of the polymer microspheres, but their effects on the porous parts are different. The glass microspheres in the polymer matrix can enhance the mechanical strength and specific modulus of the product, while the thermal expansion microspheres lead to the opposite result [132,133]. In fact, the latter is an in situ foaming method, similar to doping chemical foaming agents [134,135,136]. In contrast, micro-extrusion stacking and porous structure formation do not occur simultaneously in post-batch foaming [137]. Firstly, solid parts are printed by an FDM printer, and then foam parts are obtained through physical foaming (such as temperature rise foaming or pressure release foaming). This method can obtain foam with a lower density and a more uniform and dense cell structure, but it is difficult to control the dimensional accuracy of the parts during the foaming process [138]. The last method for preparing porous parts through micro-extrusion stacking is ordered cell unit printing. FDM itself is controlled by a program and can achieve high-precision printing. Therefore, it is possible to artificially design models with porous structures and print them into porous parts, even though such bubbles are at the millimeter level. They mostly have ordered cell units and are used to study the differences in response to external forces with different unit shapes and sizes [139,140].

### 4.2. Characteristics of the Parts

**Figure 12:**
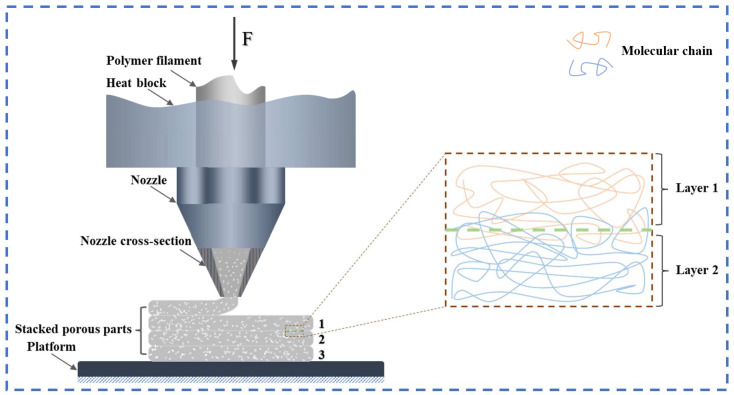
Micro-extrusion foam stacking and the plasticization effect of CO_2_ promote the migration and entanglement of polymer chains, thereby enhancing the bonding strength between strands.

The method of micro-extrusion foam stacking compensates for the shortcomings of FDM technology, which can only prepare macroscopic millimeter-sized pores and makes it difficult to achieve microporous structures [141]. This is because the millimeter-level nozzle diameter limits the forming accuracy. On the other hand, FDM is formed through the bonding mechanism between micro-extruded strands, which results in poor adhesion between strands due to the existence of the time before and after the relationship of the printing layer, and the mechanical properties of the part are significantly anisotropic [142,143]. From a microscopic perspective, in order to improve the adhesion between the micro-extruded strands, diffusion, and entanglement between molecular chains should be promoted, that is, to increase the mobility of the molecular chains. Zhou et al. [144] prepared PEI porous parts using the micro-extrusion foam stacking method and conducted tensile tests. They found that the bonding strength between the strands of foamed PEI parts (42.8 MPa) was significantly higher than that of non-foamed (22.6 MPa), and they claimed that this was due to the supercritical fluid (CO_2_) dissolved in the polymer filament acting as a plasticizer in the micro-extrusion process, which increased the migration rate of the molecular chains. As shown in Figure 12, the presence of CO_2_ improves the diffusion ability of polymer molecular chains, and the foaming expansion of extruded strands increases the contact area and pressure between the stacked layers, which makes the molecular chain entanglement between the stacked layers more compact [102]. Previous studies have shown that CO_2_ can serve as a plasticizer to reduce the glass transition temperature (T_g_) and the viscosity of polymer matrices [145,146,147], providing support for the above analysis.

**Figure 13:**
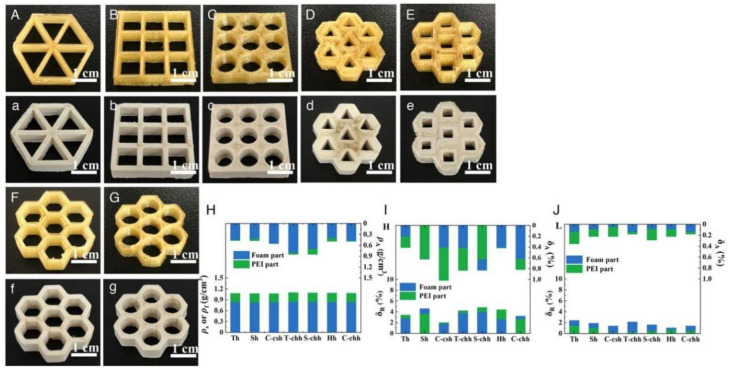
Porous PEI parts with various lattice structures and their corresponding printing accuracy [148]. Optical photographs of (**A**–**G**) PEI honeycomb structures and (**a**–**g**) foam PEI honeycomb structures. (**H**) Density of PEI parts and foam PEI parts. (**I**,**J**) Relative accuracy (*δ_R_*) and absolute accuracy (*δ_A_*) of PEI parts and foam PEI parts stacked along different printing directions.

**Figure 14:**
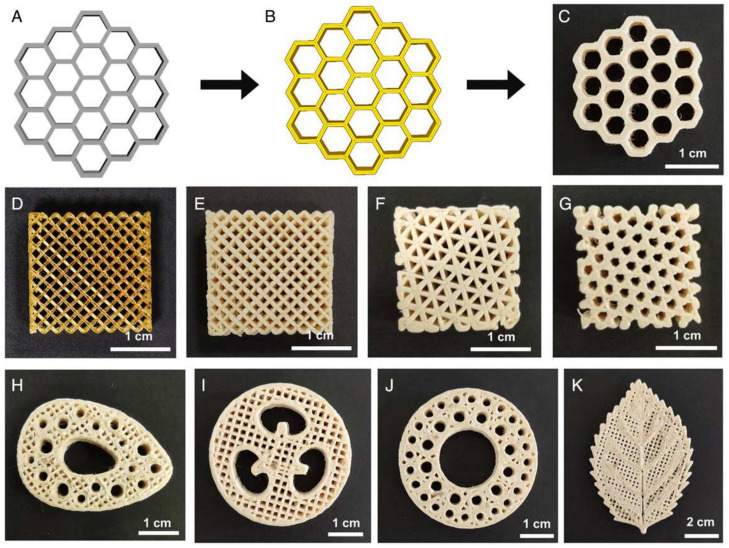
Diversified PEI bionic hierarchical porous parts. (**A**) Design a honeycomb model. (**B**) Software slicing to determine the printing path. (**C**) The physical object obtained by micro-extrusion foam stacking, honeycomb, (**D**) grid scaffold (un-foamed), (**E**) grid scaffold (foamed), (**F**) triangle scaffold, (**G**) hexagonal scaffold, (**H**) bone, (**I**) loofah, (**J**) bamboo, and (**K**) leaf [126].

Furthermore, inevitably, un-foamed extruded strands are stacked in a cylindrical shape, with gaps between them [31], which is a periodic defect. The fact that strands will experience expansion after foaming as well as a decrease in melt viscosity enables us to greatly reduce the intrinsic periodic defect. However, this foaming expansion phenomenon can also reduce the precision of extrusion stacking molding, and the accuracy is also affected by multiple factors. In response to this issue, Zhai et al. [148] investigated the influence of factors such as the resolution layer thickness, desorption time, and lattice structure on the dimensional accuracy of porous parts. The study revealed that foamed PEI parts exhibited higher dimensional accuracy in the external regions, with low and similar dimensional deviations along both the horizontal and vertical directions. However, the internal regions of foamed PEI parts showed poorer printing accuracy. With increasing desorption time, the printing accuracy improved. They also examined the impact of the layer thickness and lattice structure on the dimensional accuracy of printed parts. The results indicated that MEF exhibited excellent printing performance and controllable printing accuracy. Porous PEI parts with various lattice structures exhibited good printing performance, consistent coloration, uniform dimensions, and rare surface defects (Figure 13). To enhance the internal dimensional accuracy of porous parts, a precision correction method based on nozzle expansion was proposed. This correction method considered the expansion of the nozzle during the printing process and adjusted the printing parameters accordingly. Building upon this approach, their team produced numerous PEI biomimetic-graded porous structures (Figure 14), demonstrating the potential for highly adjustable control of porous morphology through the in situ stacking capabilities of MEF [106].

### 4.3. Different Material Systems

Micro-extrusion foam stacking, as a novel additive manufacturing method, merges supercritical fluid foaming with extrusion deposition to generate controllable customized 3D structures with both macroscopic and microscopic pores. This innovative approach was first reported by Marascio et al. [106]. In recent years, this solvent-free environmentally friendly process has been validated for use in various materials, and we summarize it in Table 2. The process and equipment of physical foaming are simple and applicable to a wide range of polymer systems [84,134,149,150], which means that the micro-extrusion foam stacking process is still expected to be extended to other material systems to meet different application requirements.

## 5. Summary and Outlooks of MEF

This review provides a comprehensive summary of the process steps, principles, and current developmental status of utilizing MEF as a novel method for preparing porous fibers and parts. MEF, as a physical foaming technology, integrates with FDM. It involves the conversion of raw materials into a filament form, saturation with high-pressure fluid, and the generation of a porous structure through micro-extrusion melt foaming. MEF emerges as an effective approach to balancing production efficiency and cost while aligning with the developmental trend of environmental protection themes. Crucially, MEF is not a separate but an interrelated relationship in the preparation of porous fibers and porous parts. The first three steps of the MEF process are necessary and uniform, with only the fourth step leading to the generation of two distinct product endpoints. Although the current application of material systems for preparing porous fibers is limited, various material systems, including engineering plastics, ordinary plastics, and polymer elastomers, have been successfully employed in the manufacturing of porous parts. This means that such a material system is likely to also be used to prepare porous fibers and will have good results.

Whether it is from the perspective of porous fibers or porous parts, MEF has a forward trend. After all, the development of novel methods to prepare porous fibers and the combination of additive manufacturing and physical foaming are currently important research directions. By reviewing the process, principle, and development status of MEF, the following eight advantages can be summarized: (1) desolvated physical foaming, green and environmental protection, (2) high efficiency, (3) lightweight, (4) combination FDM, the geometric shape is not limited, (5) the mechanical strength of porous parts is improved, (6) the size and density of cells are affected by many factors and are controllable, (7) suitable for multi-material systems and the future research space is large, (8) the applications of thermal insulation fabrics, medical stents, catalysis, and adsorption have initially taken shape, and are expected to expand to wearables, drug carriers, osmotic separation, sensors, energy storage, and other fields in the future. However, the existing problems of MEF must also be faced squarely. For example, some materials have low porosity and cannot micro-extrude and foam for a long time, because the supercritical fluid foaming agent loss occurs too fast in the polymer filament under the air atmosphere. The diffusion capacity of the blowing agent can be reduced by polymer material modifications, or more simply, the processing time can be extended by changing the external environment, such as by lowering the ambient temperature. In addition, MEF is a process of heating, foaming, and cooling, so establishing a real and effective temperature field simulation for the research system will be very beneficial to the foaming results.

## Figures and Tables

**Table 1:** The characteristics of the four foaming techniques.

Foaming Technology	Foaming Method	Raw Material	State of Polymer before Foaming	State of Polymer during Foaming	Cell Morphology	Expansion	Morphology of Foaming Materials
MEF technology	Rapid heating	Polymer filament	Glassy state	Viscous state	Uniform cellular structure	Low	Foamed fibers or foamed complex 3D components
Continuous extrusion foaming technology	Pressure quenching	Polymer pellets	Viscous state	Viscous state	Non-uniform cellular morphology	Low	Foamed fibers or foamed sheets
Injection molding foaming technology	Pressure quenching	Polymer pellets	Viscous state	Viscous state or high-elastic state	Uniform cellular structure	High	Foamed profiles
Batch foaming technology	Pressure quenching or rapid heating	Polymer pellets or sheets or profiles	Glassy state or high-elastic state	High-elastic state	Uniform closed-cell structure	Very high	Foamed beads or foamed sheets or foamed profiles

**Table 2:** Material systems that have been used for micro-extrusion foam stacking, including PLA [106,151,152,153], PEI [102,126,144,148], ABS [154], and TPU [155,156,157].

Micro-Extrusion Foam Stacking			
Material		Motivation	Year
Polylactic acid (PLA)	PLA	Mechanism research	2017
	PLA/βTCP, PLCA	Application of tissue engineering	2017
	PLA/CFs	Enhancement of mechanical properties	2021
	PLA	Mechanism research	2022
	PLA	Printing parameter optimization	2023
Polyetherimide (PEI)		Production of hierarchical porous parts	2020
		Dimensional accuracy research	2021
		Production of biomimetic structural parts	2021
		Research on interfacial adhesion enhancement	2023
Acrylonitrile butadiene styrene (ABS)		Lightweight	2021
Thermoplastic polyurethane (TPU)		Production of porous heterostructures	2022
		Application of tissue engineering	2022
		Study on properties of hierarchical cellular foam	2023

## Data Availability

The data are available through the reported references.
